# Cigarette Smoking and Survival of Patients with Non-Melanoma Skin Cancer: A Systematic Literature Review and Meta-Analysis

**DOI:** 10.3390/cancers17223670

**Published:** 2025-11-15

**Authors:** Chiara Andreon, Aurora Gaeta, Maddalena Carretti, Alice Graziani, Giulio Tosti, Chiara Doccioli, Maristella Saponara, Giuseppe Gorini, Mariano Suppa, Elisa Di Maggio, Sara Gandini, Saverio Caini

**Affiliations:** 1 Department of Experimental Oncology, IEO European Institute of Oncology IRCCS, 20141 Milan, Italy; 2 Department of Statistics and Quantitative Methods, University of Milano-Bicocca, 20126 Milan, Italy; 3 Medical Specialization School of Hygiene and Preventive Medicine, University of Florence, 50134 Florence, Italy; 4 Dermato-Oncology Unit, IEO European Institute of Oncology IRCCS, 20141 Milan, Italy; giulio.tosti@ieo.it; 5 Clinical Epidemiology Unit, Institute for Cancer Research, Prevention, and Clinical Network (ISPRO), 50139 Florence, Italye.dimaggio@ispro.toscana.it (E.D.M.); 6 Division of Melanoma and Sarcoma Medical Treatment, IEO European Institute of Oncology IRCCS, 20141 Milan, Italy; 7 Department of Dermatology, Hôpital Erasme, Université Libre de Bruxelles, 1070 Brussels, Belgium; 8 Cancer Risk Factors and Lifestyle Epidemiology Unit, Institute for Cancer Research, Prevention, and Clinical Network (ISPRO), 50139 Florence, Italy; s.caini@ispro.toscana.it

**Keywords:** non-melanoma skin cancer, basal cell cancer, squamous cell cancer, cigarette smoking, survival, literature review, meta-analysis

## Abstract

Background: Non-melanoma skin cancer (NMSC) is the most frequent cancer in fair-skinned populations and represents a growing public health concern due to its impact in terms of morbidity and treatment costs. While some meta-analyses have investigated cigarette smoking as a risk factor for NMSC, less is known about its prognostic implications in patients with NMSC. This systematic review and meta-analysis aims to fill this gap by assessing the association between smoking habits and survival in patients with NMSC. Methods: A systematic search was conducted in PubMed and EMBASE up to 25 February 2025, to identify prospective studies of patients with histologically confirmed NMSC that evaluated the association between smoking habits and survival. Study-specific hazard ratios (HRs) and corresponding 95% confidence intervals (CIs) were pooled using random effects meta-analysis models. Results: A total of five studies published between 2015 and 2022 were included. The meta-analysis revealed that being a current or ever smoker at diagnosis was associated with a worse overall survival (summary HR 2.42, 95% CI 1.91–3.06). A similar result was observed when smoking exposure was assessed in terms of pack-years or number of cigarettes per day (summary HR 2.44, 95% CI 2.02–2.93). Conclusions: Our findings indicate that cigarette smoking is a negative prognostic factor in these patients, despite the generally excellent prognosis of NMSC. It is reasonable to assume that this unfavourable effect is largely due to the increased risk of developing other life-threatening conditions, in which smoking plays a causal role. These results underscore the clinical relevance of systematically integrating smoking cessation counselling into the routine management of patients with NMSC.

## Simple Summary

There is still limited evidence on whether cigarette smoking may affect the prognosis of patients with non-melanoma skin cancer (NMSC). Here, we conducted a systematic review and meta-analysis of published reports in Pubmed and Embase to identify prospective studies of patients with histologically confirmed NMSC that evaluated the association between smoking habits and survival. The search was updated to February 2025. Based on five eligible articles published between 2015 and 2022, we found that the overall survival in patients with NMSC is negatively affected by current or ever smoking, as well as by the number of cigarettes smoked per day and the number of pack-years smoked in one’s lifetime. These results indicate that cigarette smoking is a negative prognostic factor in this patient population and underscore the importance of systematically integrating smoking cessation counselling into the routine management of patients with NMSC.

## 1. Introduction

The term “non-melanoma skin cancer” (NMSC) traditionally refers to skin cancers that arise from the keratinocytes of the epidermis and mainly includes basal cell carcinoma (BCC) and squamous cell carcinoma (SCC), with the former accounting for roughly three-quarters of all NMSC diagnoses. Although less common, SCC is more aggressive than BCC, as it grows more rapidly and is more likely to metastasize; as a consequence, SCC is responsible for the majority of NMSC-related mortality [1,2]. Globally, NMSC is the most common diagnosed malignancy among Caucasians: according to the 2022 GLOBOCAN update, roughly 1.23 million new NMSC cases and nearly 70,000 related deaths occurred worldwide, placing the disease fifth among all cancers for incidence, yet its true burden is underestimated because of deficient reporting [3]. Between 1990 and 2021, global incidence in people aged ≥60 years almost tripled; in particular, age-standardized incidence for both BCC and SCC rose significantly, with the sharpest increases in high-income countries [3]. Regarding sex differences, a persistent male excess for squamous cell disease is observed, possibly due to greater cumulative sun exposure and less frequent adoption of skin protection practices. Projections suggest that, even if incidence rates reach a plateau, the absolute case load and disability burden will keep growing through 2050 [4].

Within this framework, NMSC has emerged as a health concern of growing importance, due to its burden in terms of both morbidity and treatment costs [5]. In particular, the increasing age of the general population, combined with greater sun exposure, represents the main factor contributing to the clinical and economic burden associated with NMSC. These tumours are characterized by multiple risk factors, both endogenous (Fitzpatrick skin phototypes I-III, fair skin and blue eyes, dysplastic nevi, family history, and genetic conditions such as oculocutaneous albinism and xeroderma pigmentosum) and exogenous (ultraviolet radiation exposure, low sun protection, history of sunburn, and HPV infection) [6]. In particular, SCC risk is more typically associated with cumulative lifetime sun exposure, while intermittent and intense UV exposure increases the risk of BCC [7]. Occupational exposure to UV radiation represents a major risk factor for NMSC occurrence among outdoor workers: the World Health Organization (WHO) and the International Labour Organization (ILO) have estimated that nearly 30% of global NMSC deaths in 2019 were attributed to solar UV exposure among outdoor workers, and the attributable mortality has almost doubled since 2000 [8]. Other occupational risk factors include chronic skin exposure to inorganic arsenic, ionizing radiation, mineral oils, and pesticides all of which seem to be linked to an increased NMSC incidence, particularly SCC [9,10].

Cigarette smoking increases the risk of cancers at multiple body sites; moreover, being an active smoker at diagnosis generally has an adverse effect on the prognosis of cancer patients. There are many mechanisms through which cigarette smoking can negatively impact on the survival of cancer patients, e.g., by promoting cancer cell survival and growth, favouring angiogenesis and subsequent distant metastases, creating an immunosuppressive microenvironment, and interfering with the efficacy of cancer treatments [11,12]. In the context of NMSC, several meta-analyses have reported smoking as a risk factor for SCC, while evidence regarding BCC remains controversial, with some recent analyses even suggesting an apparent inverse association between current smoking and BCC risk [13,14,15,16,17]. Much less is known about the prognostic implications of smoking history and intensity among patients diagnosed with NMSC, and whether any difference exists between BCC and SCC. In particular, unlike for most other cancer types, no systematic review to date has ever tried to summarize the existing evidence on the association between smoking and NMSC prognosis. Thus, the present systematic review and meta-analysis aims to address this gap by assessing the association between smoking habits and overall survival and other clinical endpoints in patients with NMSC, and by examining whether greater cumulative exposure to smoking is associated with differences in prognosis.

## 2. Materials and Methods

### 2.1. Literature Search, Article Selection, and Data Extraction

The review protocol was preregistered on the Prospero platform (https://www.crd.york.ac.uk/PROSPERO/view/CRD420251110987, accessed on 24 September 2025). Every phase—study design, execution, and manuscript preparation—followed the MOOSE recommendations for meta-analyses of observational studies and adhered to the PRISMA reporting checklist (available as File S1). The research questions were formulated using the following PECO structure. The study population consisted of patients with histologically confirmed NMSC (basal cell or squamous cell carcinoma of the skin). The exposure of interest was cigarette smoking status (current, former, never) and history (ever, never), any measure of frequency (e.g., expressed as pack-years or cigarettes per day), as well as other information such as the age at smoking initiation and cessation, the time between cessation and NMSC diagnosis (for former smokers), and others. When dose–response analyses were available, heavier smokers (e.g., higher amount of cigarettes/day for current smokers, or higher cumulative pack-years for current and former smokers) were compared with lighter or never smokers. The primary outcome of interest was overall survival (OS), defined as the time from histological diagnosis (or study entry for treated cohorts) to death from any cause. Secondary outcomes encompassed disease-specific survival (DSS), disease-free survival (DFS), and progression-free survival (PFS), depending on availability.

The literature search was conducted in PUBMED and EMBASE on 25th February, 2025, by using the search strings reported in File S2. Of note, the search string was deliberately constructed to be highly sensitive in order not to miss any existing eligible articles, which were expected to be limited in number. Both databases were searched until inception, and no language or geographical restrictions were applied (when necessary, the abstract and text of non-English articles were translated into English using AI-based online tools to make a final decision on eligibility). After removing duplicates, all articles were initially assessed based on their title and abstracts; those judged as potentially eligible for inclusion were then read in full text to verify inclusion and exclusion criteria. The selection of articles was conducted independently by two researchers (AG and MC), and any disagreement was resolved by arbitration by a third, senior researcher (SC).

We included in this review and meta-analysis articles that followed up patients with histologically confirmed NMSC (BCC and/or SCC) after diagnosis and evaluated the association of smoking habits with OS or another outcome of interest (as detailed above). To be eligible, an article had to provide, or allow the calculation of, a quantitative effect estimate for at least one pre-specified comparison (e.g., current, former, or ever vs. never smokers, high vs. low number of cumulative pack-years, etc.), expressed as a hazard ratio (HR; or subdistribution hazard ratio, sHR, in case of competing risks survival analysis) with its 95% confidence intervals (CIs).

Study quality was assessed using the Quality in Prognosis Studies (QUIPS) tool [18].

### 2.2. Statistical Analysis

To assess different smoking habits, two meta-analyses were conducted, the first focusing on patients’ smoking status/history (a), while the second focused on pack-years/cigarettes per day (b). We log-transformed the study-specific HRs and 95% CI and calculated the corresponding variance [19]. In order to obtain the summary HR, we fitted random-effect models with restricted maximum likelihood estimations (this choice was motivated by the large diversity between studies in terms of design and other characteristics, which made the use of fixed-effect models questionable) [20]. To consider the dependence of estimates on the same study, we adapted a hierarchical model, where necessary [21]. A pre-planned subgroup analysis was conducted by NMSC subtype, as well as a sensitivity analysis comparing former vs. never smokers and current vs. never smokers.

The I2 statistic was calculated to assess the heterogeneity of HRs between the studies. Values of the I2 statistics above 50% pointed out high heterogeneity. Publication bias was checked graphically by funnel plots and formally by Egger’s tests [22]. Statistical analyses were conducted using R software, version 4.5.1. All tests were two sided and statistical significance was set at *p*-values below 0.05.

## 3. Results

**Figure 1:**
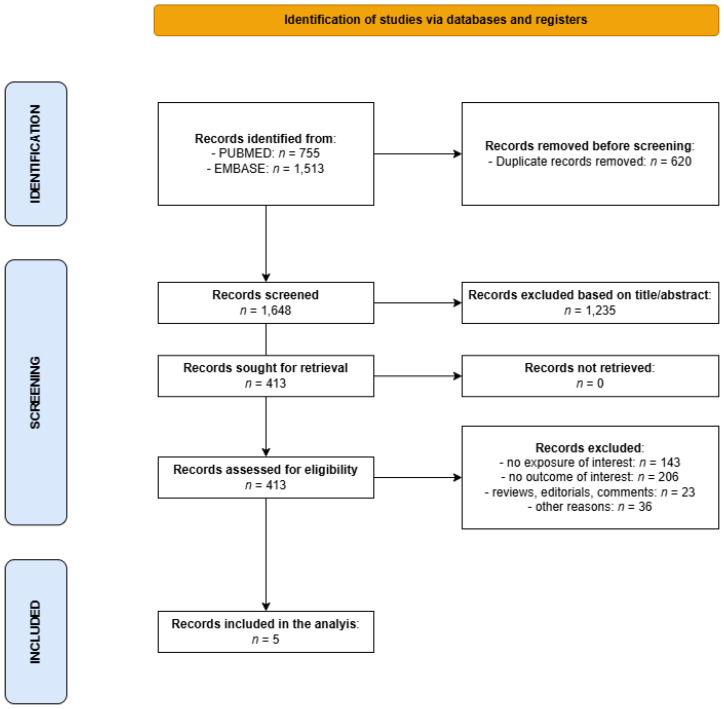
Flowchart for the selection of studies on the association between cigarette smoking and survival of patients with NMSC that were included in the present meta-analysis.

The literature search returned 2268 entries, of which 1648 remained for screening after deduplication. Of these, 1235 were discarded based on their title and abstract, and 413 were retrieved for full-text reading, of which 408 were discarded for not meeting the inclusion criteria (the main reason for exclusion was the failure to include the exposure or an endpoint of interest) (Figure 1).

Finally, five independent studies published between 2015 and 2022 were included in the systematic review [23,24,25,26,27], described in Table 1.

These studies were conducted in the USA (*n* = 3), Taiwan (*n* = 1), and Hong Kong, China (*n* = 1), and provided estimates for SCC only (*n* = 3; for SCC of the lip, head and neck, or any site, *n* = 1 each), separately for BCC and SCC (*n* = 1), or for NMSC overall with no separate results by subtype (*n* = 1). The studies varied considerably in terms of mean/median age at diagnosis (ranging from 59.4 to 84.0 years), sex (male % ranged from 45.2% to 88.4%), and median length of follow-up between 1.5 and 13.0 years. Smoking exposure at diagnosis was measured by assessing smoking history (ever vs. never, *n* = 2), smoking status (current vs. former vs. never, *n* = 2), the number of cigarettes smoked per day (*n* = 1), or the cumulative number of pack-years (*n* = 2). In one study, an association between years since cessation and survival was also estimated. No study investigated the prognostic effect of changes in smoking habits that occurred after diagnosis. The effect of smoking habits was evaluated in relation to overall survival in all studies except Tseng et al. (2017) [24], which focused on DSS and DFS.

Study quality was judged as generally fair, with most studies having a low or moderate risk of bias in all domains of the QUIPS tool (File S3). The susceptibility to bias was judged as “moderate” for all included studies in the “Study attrition” domain due to the failure to provide detailed information on the proportion of patients lost to follow-up, and in the “Prognostic factor measurement” domain since all studies relied on self-reporting instead of using more reliable methods such as monitoring exhaled carbon monoxide or measuring urinary or salivary cotinine.

**Figure 2:**
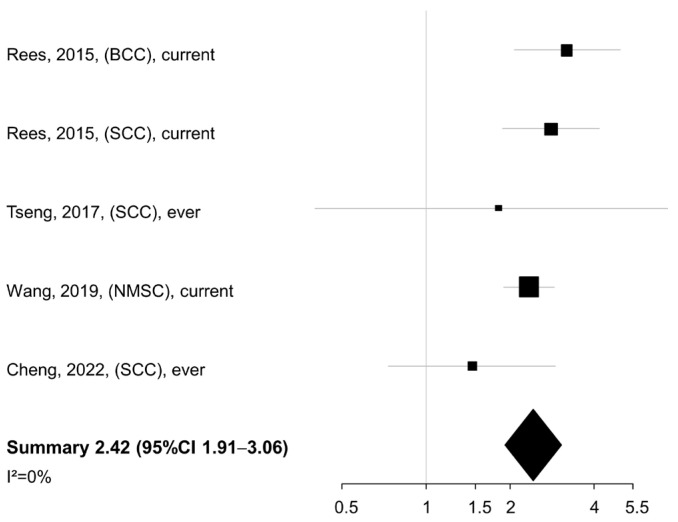
Meta-analysis of the association between smoking status/history (current/ever vs. never smokers) and overall/cancer-specific survival of patients with non-melanoma skin cancer. BCC: basal cell cancer. SCC: squamous cell cancer. NMSC: non-melanoma skin cancer. CI: confidence interval [23,24,26,27].

Being a current or ever smoker at diagnosis was associated with a significantly increased risk of death (summary HR 2.42, 95% CI 1.91–3.06) according to data from four studies (Figure 2).

**Figure 3:**
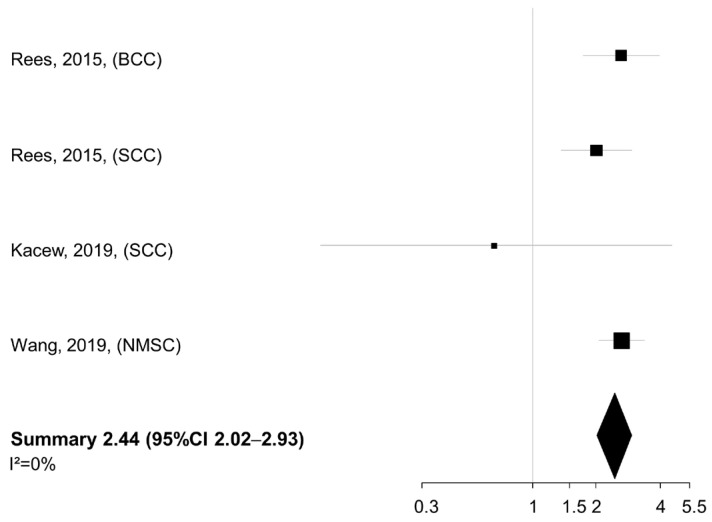
Meta-analysis of the association between smoking intensity (pack-years or cigarettes/day, highest vs. lowest category) and overall survival of patients with non-melanoma skin cancer. BCC: basal cell cancer. SCC: squamous cell cancer. NMSC: non-melanoma skin cancer. CI: confidence interval [23,25,26].

No heterogeneity was found across studies (I^2^ = 0%). When the smoking exposure was evaluated in terms of pack-years or cigarettes per day (*n* = 3 studies), a similar summary HR was detected for the comparison of NMSC patients in the highest vs. lowest category of exposure (summary HR 2.44, 95% CI 2.02–2.93), again with no between-study heterogeneity (I^2^ = 0%) (Figure 3).

In both meta-analyses (a) and (b), there was no evidence of publication bias from the Egger’s test (*p* = 0.780 and *p* = 0.175, respectively; the corresponding funnel plots are in Figures S1 and S2). In the SCC subgroup analysis, the summary HR was only slightly reduced compared to the overall analysis (summary HR 2.15, 95% CI 1.28–3.61) (Figure S3). In additional sensitivity analyses, the summary HR was 2.57 for the comparison of current vs. never smokers at diagnosis (95% CI 2.05–3.24) (Figure S4), and 1.23 when comparing former vs. never smokers (95% CI 1.01–1.51) (Figure S5). No indication of publication bias emerged in any of these subgroup analyses (*p*-value 0.547, 0.132, and 0.479, respectively).

## 4. Discussion

This is the first systematic review and meta-analysis that summarizes the evidence on the prognostic impact of cigarette smoking among patients with NMSC. A very low case-fatality rate generally characterizes skin cancers originating from keratinocytes (BCC and SCC), but, in some patients, the tumour can metastasize and cause the patient’s death, albeit with very different rates: BCC much more rarely than SCC [28,29]. It is widely recognized that most known prognostic factors for NMSC are tumour-related. Among patients with BCC, lower prognosis is related to aggressive histological subtype, perineural invasion, local destruction, and secondary recurrence, with the risk of recurrence being influenced by tumour location and size, infiltrative or positive surgical margins, immunosuppression, and previous radiotherapy [30,31,32,33]. For SCC, poorer prognosis is influenced by tumour size and location, tumour thickness/depth of invasion, perineural invasion, low histological differentiation, desmoplasia, and growth rate [34]. While these tumour-related risk factors remain of primary importance but are generally less modifiable by lifestyle, addressing smoking habits is crucial as these habits are potentially modifiable: thus, the scientific question we tried to answer by conducting this meta-analysis has immediate clinical implications for treating physicians.

Studies eligible for inclusion were limited in number, but remarkably consistent in showing that cigarette smoking represents a negative prognostic factor among patients with NMSC (including in subgroup analyses, e.g., limited to SCC), despite some variability between studies in how smoking habits were recorded. Considering that the prognosis of NMSC is generally excellent, and all but one of the included studies considered overall instead of cancer-specific survival, it appears reasonable to conclude that the negative prognostic effect of cigarette smoking in these patients may be at least partly due to the risk of developing one of the many life-threatening diseases in whose pathogenesis smoking is implicated, and, possibly, also to the occurrence of a second primary melanoma, whose incidence is known to be increased among patients with a previous NMSC. Whether a direct effect of smoking on NMSC-specific survival exists is currently difficult to establish, as most eligible studies do not provide separate association estimates for overall and cause-specific endpoints. It is noteworthy, however, that the only study to date that considered NMSC-specific survival as an endpoint of interest (that by Tseng et al. 2017 [24]) detected an over 80% increase in the risk of (disease-specific) death among ever- compared to never smokers [24]. There is extensive evidence that cigarette smoking may promote immune escape and ultimately the development of distant metastases [35,36,37,38,39,40], which in turn represents the primary mechanisms by which NMSC (particularly SCC) can cause patient death [41,42,43]. In addition, as already mentioned, cigarette smoking may favour cell growth and distant spread as well as lessen the effectiveness of systemic treatments [11,12]. Therefore, despite the lack of laboratory evidence specific to keratinocyte carcinomas and the paucity of relevant epidemiological data, it is not biologically implausible to hypothesize that exposure to cigarette smoke may also negatively impact the clinical course of NMSC, favouring local progression and/or metastatic spread.

Regardless of the potential underlying mechanisms, the clinical implications of our findings are clear, as they reinforce the notion that providing counselling and continuous support for smoking cessation should be mandatory for all healthcare professionals, even when dealing with healthy individuals or patients suffering from conditions that are clinically benign and often with limited impact on quality of life (as in the case of NMSC). Given the very high incidence of keratinocyte cancers, many patients who visit their general practitioner or dermatologist with this condition might be otherwise healthy individuals except for being active smokers. For this reason, smoking cessation support should be an integral part of the routine management of patients with NMSCs who are active smokers at the time of diagnosis, and any contact with these patients should be regarded as a potential “teaching moment” not to be missed to induce healthy changes in behaviours, including smoking cessation.

The main strength of our work lies in providing the first comprehensive review of the available evidence regarding the impact of cigarette smoking on the prognosis of patients with NMSC. Heterogeneity across study-specific association estimates was negligible, and there was no evidence of publication bias; most included studies were of fair-to-good quality. The evidence provided by our meta-analysis is, however, weakened by several limitations, among which the most obvious one is the very low number of studies that were found to be eligible for inclusion. While conducting a systematic literature review is always worthwhile, for instance to address any gaps in the available evidence on a given topic, a limited number of eligible studies (as is the case here) makes it inevitably difficult to draw firm conclusions and conduct more in-depth analyses (including subgroup analyses and meta-regression aimed at assessing the impact of the study design as well as patients’ and tumour’s characteristics on the summary estimates). In addition to the limited sample size, other limitations need to be acknowledged. Smoking status was recorded in all studies at diagnosis and referred to either smoking status or history. In contrast, no study reported on the prognostic effect of smoking cessation among patients who were current smokers at diagnosis. While promoting smoking cessation is an objective that is always worth pursuing, the lack of longitudinal studies tracking changes in smoking behaviour post-diagnosis limits our ability to evaluate whether and by how much post-diagnosis smoking cessation would improve survival among these patients. Past and current exposure to cigarette smoking was assessed through self-reporting by patients in all included studies: this might make the studies more vulnerable to reporting bias and misclassification, as participants might inaccurately recall (e.g., underreport) their smoking habits. Moreover, there was no consistent threshold for identifying “heavier” vs. “lighter” smokers (either using cigarettes/day or pack-years), and second-hand smoking was not reported in any of the included studies. BCC and SCC were merged together in most included studies, which may represent a major source of bias given the difference in case-fatality rates between these two skin cancer types. Finally, it was not possible to investigate whether the prognostic impact of smoking habits on the survival of patients with NMSC varies according to tumour stage and treatment modalities, due to the scarcity of information on these aspects in most included studies (as well as the limited number of studies included in the meta-analysis).

## 5. Conclusions

In conclusion, although the studies included in this meta-analysis only considered smoking habits at diagnosis (and not post-diagnosis cessation), our findings support the importance of systematically integrating smoking cessation counselling into the routine management of patients with NMSC, and of strengthening the role of healthcare professionals in supporting smoking cessation efforts. Such an approach may improve general survival among NMSC patients (especially due to the risk of other smoking-related diseases, e.g., cardiovascular, pulmonary, and other cancers) and increase the overall effectiveness of NMSC management strategies. Importantly, future studies should focus on the prognostic impact of post-diagnosis smoking cessation among patients with NMSC who are active smokers at diagnosis, as this would more clearly translate into clinical recommendations for treating physicians.

## Figures and Tables

**Table 1:** Selected characteristics and main results of the studies included in the systematic review and meta-analysis on the association between smoking habits and prognosis of patients with non-melanoma skin cancer (NMSC).

Author, Year	Country	*N* Patients by Type and Site	Demographics	Follow-Up	Exposure	Comparison	HR (95% CI) ^1^
Rees, 2015 [23]	USA	1363 BCC + 880 SCC, any site	50.1% (BCC) and 61.4% (SCC) men mean/median age not reported	mean 10.3 (BCC) and 9.8 (SCC) years	smoking status	former vs. never smoker	BCC 1.27 (0.87–1.83), SCC 0.94 (0.67–1.32)
						current vs. never smoker	BCC 3.19 (2.06–4.94), SCC 2.80 (1.88–4.18)
					pack-years	0.1–20 vs. never smoker	BCC 0.88 (0.55–1.42), SCC 0.73 (0.47–1.13)
						20.1–40 vs. never smoker	BCC 1.83 (1.18–2.85), SCC 1.25 (0.82–1.90)
						>40 vs. never smoker	BCC 2.63 (1.74–3.97), SCC 2.01 (1.37–2.93)
Tseng, 2017 ^2^ [24]	Taiwan	112 SCC, lip	88.4% men mean age 59.4 years	not reported	smoking history	ever vs. never smoker	DSS 1.82 (0.40–8.23) DFS 1.09 (0.41–2.95)
Kacew, 2019 [25]	USA	33 SCC, any site	81.8% men median age 68 years	median 17.8 months	pack-years	≥10 vs. <10 or never smoker	0.66 (0.10–4.56)
Wang, 2019 [26]	USA	6286 NMSC, any site	not reported	median 13 years	smoking status	former vs. never smoker	1.33 (1.18–1.51)
						current vs. never smoker	2.34 (1.90–2.88)
					cigarettes/day (current smokers)	<10 vs. never smoker	2.01 (1.51–2.67)
						≥10 vs. never smoker	2.63 (2.05–3.37)
					time since cessation (former smokers)	<10 yrs vs. never smoker	1.93 (1.53–2.44)
						10–29 yrs vs. never smoker	1.66 (1.37–2.00)
						≥30 yrs vs. never smoker	1.16 (1.01–1.33)
Cheng, 2022 [27]	Hong Kong, China	73 SCC, head and neck	45.2% men median age 84 years	median 44 months	smoking history	ever vs. never smoker	1.46 (0.73–2.90)

HR: hazard ratio. CI: confidence interval. BCC: basal cell cancer. SCC: squamous cell cancer. NMSC: non-melanoma skin cancer. DSS: disease-specific survival. DFS: disease-free survival. ^1^ We reported the HR and 95% CI for the association between smoking status, history, or intensity (as detailed in the column “Exposure”) and the overall survival (OS) of all NMSC patients included in the study; when not available, we reported association estimates separately for patients with BCC or SCC, or for other clinical endpoints (DSS and DFS in the study by Tseng et al., 2017 [24]). ^2^ The study by Tseng et al. (2017) [24] also included 21 BCC, but no survival data were reported for these patients.

## Data Availability

No new data were created or analyzed in this study. Data sharing is not applicable to this article.

## Supplementary Materials

The following supporting information can be downloaded at https://www.mdpi.com/article/10.3390/cancers17223670/s1, File S1. PRISMA checklist. File S2. Search string. File S3. Quality assessment using the QUIPS tool. Figure S1. Funnel plots and *p*-value from Egger’s test for the meta-analyses of the association between smoking status/history (current/ever vs. never smokers) and overall/cancer-specific survival of patients with non-melanoma skin cancer. Figure S2. Funnel plots and *p*-value from Egger’s test for the meta-analyses of the association between smoking intensity (pack-years or cigarettes/day, highest vs. lowest category) and overall survival of patients with non-melanoma skin cancer. Figure S3. Subgroup meta-analysis of the association between smoking status/history (current/ever vs. never smokers) and overall/cancer-specific survival of patients with squamous cell cancer. Figure S4. Subgroup meta-analysis of the association between smoking status (current vs. never smokers) and overall/cancer-specific survival of patients with non-melanoma skin cancer; Figure S5. Subgroup meta-analysis of the association between smoking status (former vs. never smokers) and overall/cancer-specific survival of patients with non-melanoma skin cancer.

## Abbreviations

The following abbreviations are used in this manuscript:

BCC	Basal cell cancer
CI	Confidence interval
DFS	Disease-free survival
DSS	Disease-specific survival
HR	Hazard ratio
ILO	International Labour Organziation
NMSC	Non-melanoma skin cancer
OS	Overall survival
PFS	Progression-free survival
SCC	Squamous cell cancer
sHR	Subdistribution hazard ratio
WHO	World Health Organization
