# Supplementary material for: Cigarette Smoking and Survival of Patients with Non-Melanoma Skin Cancer: A Systematic Literature Review and Meta-Analysis
Source: Cancers (Basel). 2025 Nov 15;17(22):3670. doi: 10.3390/cancers17223670 (PMC12650633; doi:10.3390/cancers17223670)
Supplement: Supplementary file 1 [file cancers-17-03670-s001.zip › File S2.pdf]

PUBMED

("Cigarette Smoking"[Mesh] OR "Tobacco Products"[Mesh] OR "Tobacco Smoke Pollution"[Mesh] OR "cigarette smok*" OR "tobacco smok*" OR "smoke exposure" OR "Smoking"[Mesh] OR smoking[tiab]) AND ("Neoplasms, Basal Cell"[Mesh] OR "Neoplasms, Squamous Cell"[Mesh] OR (("non-melanom*" OR "keratinocyte*" OR "basal cell*" OR "squamous cell*") AND (cancer* OR neoplasm* OR tumor* OR tumour*))) AND ("Skin"[Mesh] OR "Skin Neoplasms"[Mesh] OR skin OR cutan* OR epithel*) AND (risk* OR incidence* OR etiolog* OR aetiolog* OR epidemiol* OR oncogen*)

EMBASE

('cigarette smoking'/syn OR 'cigarette smoking' OR 'passive smoking'/syn OR 'passive smoking' OR 'smoking'/syn OR 'smoking' OR 'smoke exposure'/syn OR 'smoke exposure') AND ('keratinocyte carcinoma'/syn OR 'keratinocyte carcinoma' OR 'non melanoma skin cancer'/syn OR 'non melanoma skin cancer' OR 'basal cell carcinoma'/syn OR 'basal cell carcinoma' OR (('squamous cell carcinoma'/syn OR 'squamous cell carcinoma' OR 'squamous cell':ti,ab OR 'basal cell':ti,ab) AND ('skin'/mj/exp OR 'skin'))) AND (risk*:ti,ab OR incidence*:ti,ab OR etiolog*:ti,ab OR aetiolog*:ti,ab OR epidemiol*:ti,ab OR oncogen*:ti,ab)
