# Supplementary figures and images for: Cigarette Smoking and Survival of Patients with Non-Melanoma Skin Cancer: A Systematic Literature Review and Meta-Analysis
Source: Cancers (Basel). 2025 Nov 15;17(22):3670. doi: 10.3390/cancers17223670 (PMC12650633; doi:10.3390/cancers17223670)

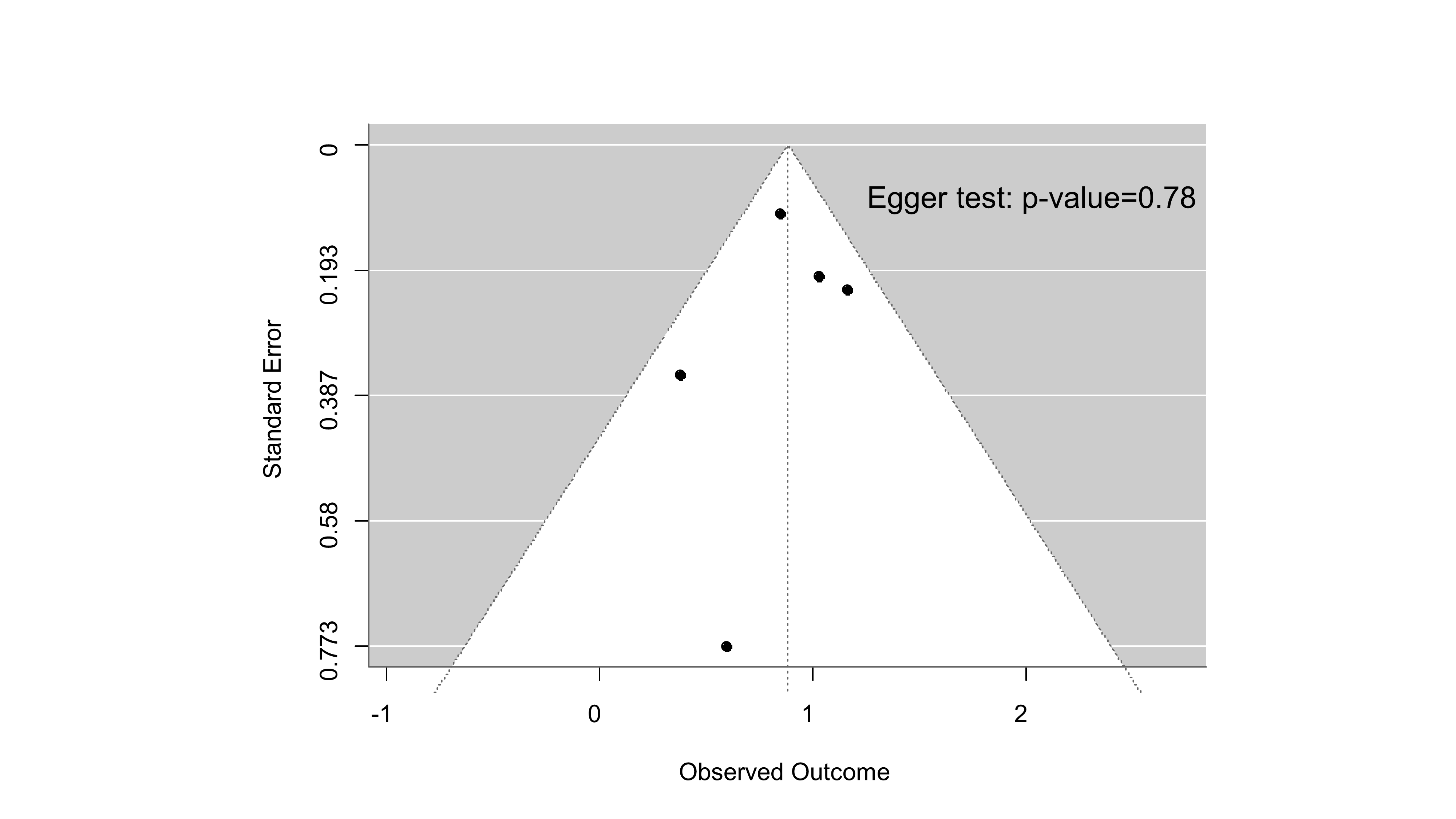

Supplement: Supplementary file 1 [file cancers-17-03670-s001.zip › Figure S1.tif]

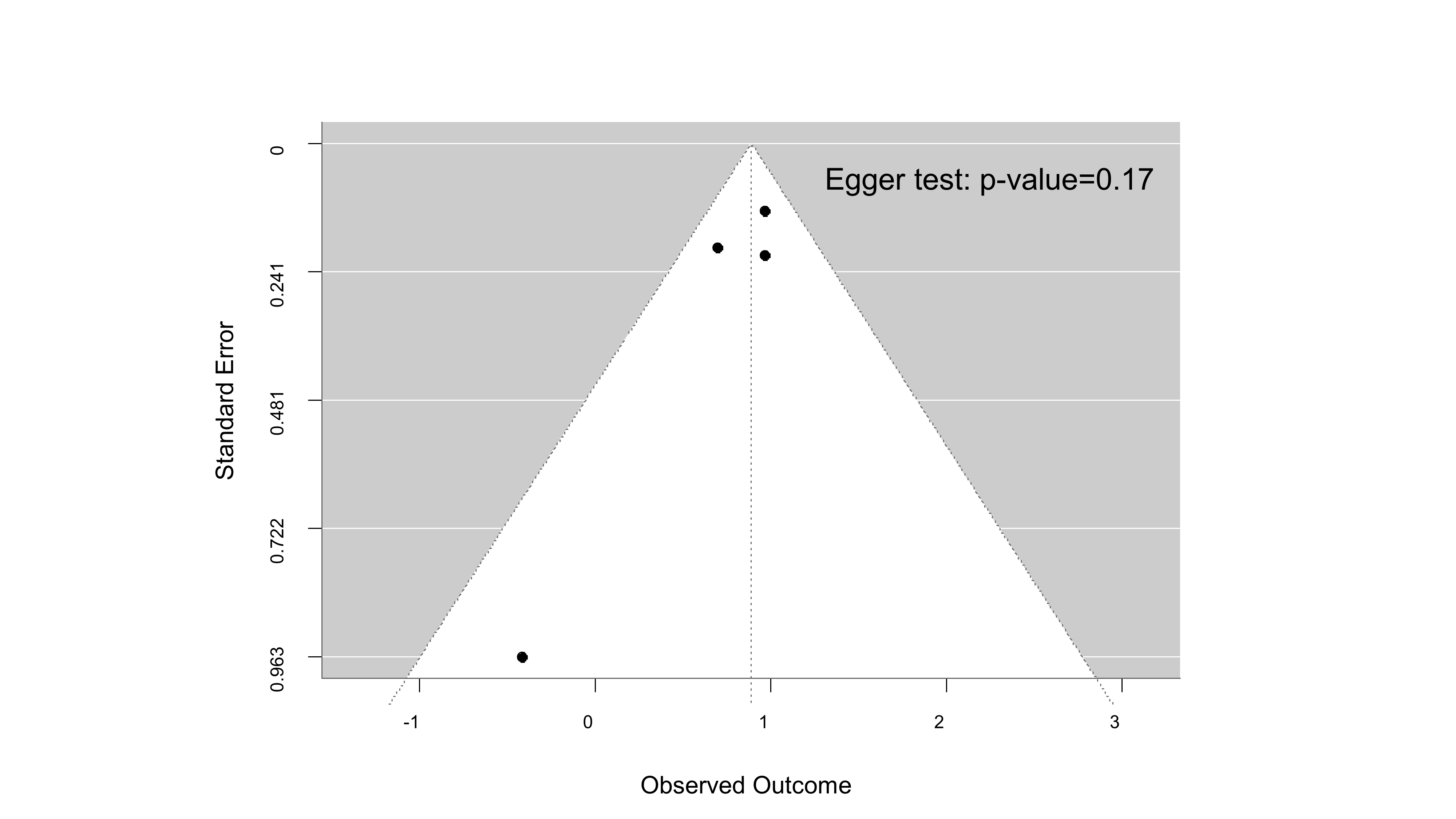

Supplement: Supplementary file 1 [file cancers-17-03670-s001.zip › Figure S2.tif]

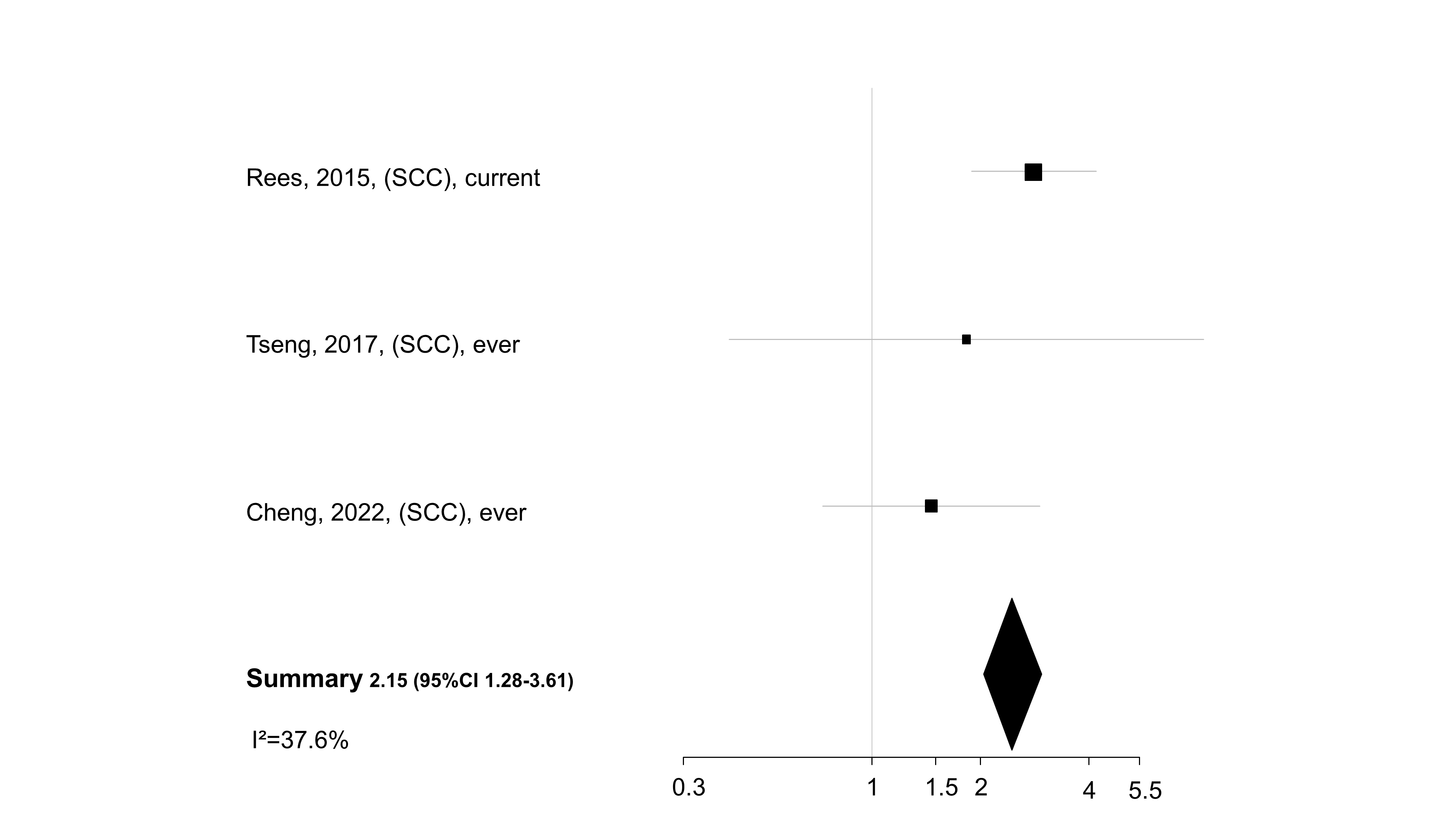

Supplement: Supplementary file 1 [file cancers-17-03670-s001.zip › Figure S3.tif]

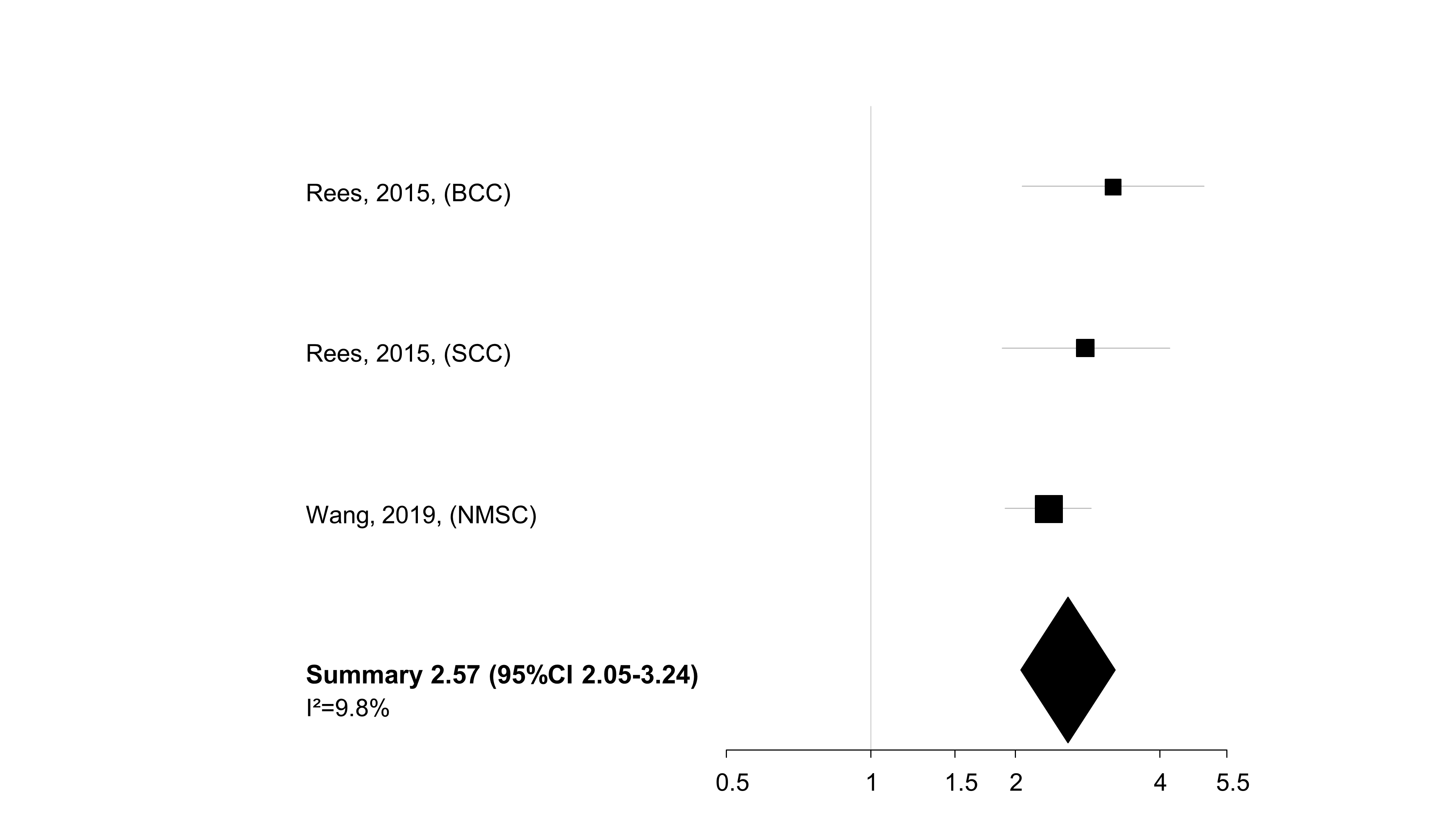

Supplement: Supplementary file 1 [file cancers-17-03670-s001.zip › Figure S4.tif]

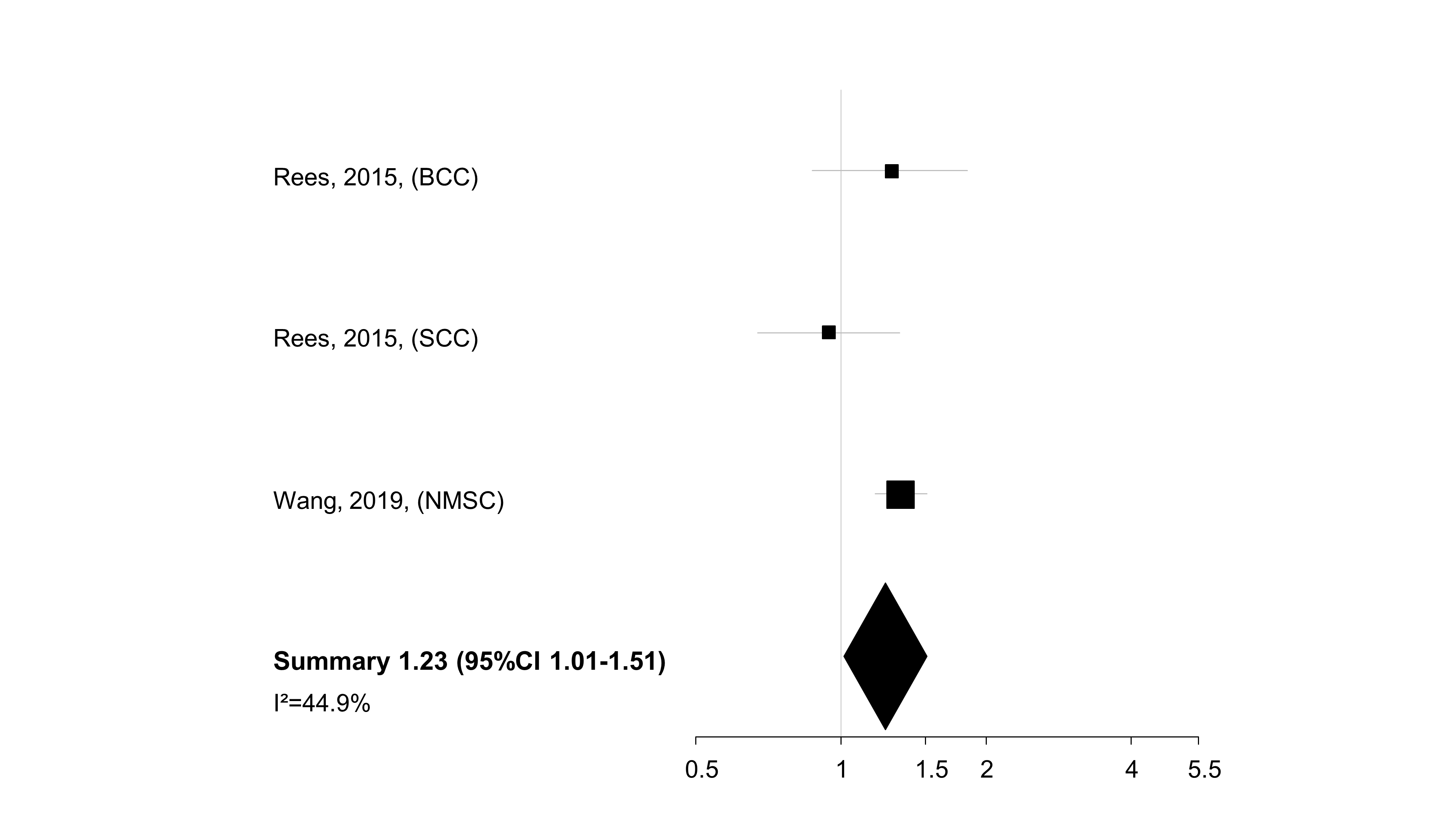

Supplement: Supplementary file 1 [file cancers-17-03670-s001.zip › Figure S5.tif]
